# Comparative mediator analysis: establishing links between mathematical thinking and creativity through digital game addiction and violent tendencies across gifted and non-gifted student groups

**DOI:** 10.3389/fpsyg.2026.1776528

**Published:** 2026-02-12

**Authors:** Zübeyde Er, Ayça Akin, Hayriye Seda Sezgin

**Affiliations:** 1 Ministry of Education, Cukurova Science and Art Center, Adana, Türkiye; 2 Department of Software Engineering, Faculty of Engineering, Antalya Belek University, Antalya, Türkiye; 3 Alumni Association, Anadolu University, Eskisehir, Türkiye

**Keywords:** Twenty first century abilities, creativity, game addiction, games, informal learning, mathematics thinking, Secondary education, violent tendencies

## Abstract

**Aim:**

In the modern era, playing digital games is a part of everyday life. This trend has led to an increased focus on the relationship between digital gaming and higher-order cognitive processes such as mathematical thinking and creativity in STEM education research. Therefore, the study investigated how mathematical thinking is directly and indirectly associated with creativity in gifted and non-gifted students through their digital game addiction behavior and tendency toward violence. Moreover, the research examined student gaming patterns as well as analyzed how these preferences modify the researched relationships.

**Methods:**

The study explored how digital game addiction and violent tendencies mediate the relationship between mathematical thinking and creativity using a primarily quantitative, sequential explanatory mixed methods design with 508 students (253 gifted and 255 non-gifted). Quantitative data were collected using the following instruments from the students: Mathematical Thinking Scale, Children's Computer Game Addiction Scale, Violence Tendency Scale, and Kaufman Domains of Creativity Scale. Then debriefing interviews were conducted with 34 volunteer students to get qualitative data. Serial mediation modeling was used to test relationships among the research variables. Moreover, an interpretative framework was applied to analyze qualitative data, focusing on students' digital gaming habits in alignment with the study's objectives.

**Results:**

The findings revealed that for the gifted student group, mathematical thinking had a stronger impact on creativity, with digital game addiction serving as a statistically significant mediator with a positive indirect pathway. In contrast, for the non-gifted student group, mathematical thinking had a moderate effect on creativity, while digital game addiction acted as a negative mediator. Although violent tendencies demonstrated statistically significant mediating effects in both groups, their magnitude was relatively small, indicating limited practical influence compared to other pathways. Additionally, gifted students preferred action, role-playing, and strategy games, whereas non-gifted students favored social-casual games. These differences in gaming behavior contributed to the distinct cognitive outcomes observed between the groups.

**Conclusion:**

The study underscores the role of digital gaming in cognitive development and highlights the need for tailored educational strategies and guided digital game use based on individual differences.

## 1 Introduction

Mathematical thinking and creativity are an integral part of cognitive development, enhancing problem-solving, analytical reasoning, and innovative thinking (Bos et al., 2013; Stolte et al., 2020). The realm of mathematical thinking involves an essential ability in abstraction, logical reasoning, and problem-solving that functions systematically in boosting cognitive abilities; mathematical thinking also interacts with creativity to enable individuals to tackle challenges in flexible and original ways (Elgrably and Leikin, 2021; Lithner, 2017; Nordby et al., 2022). These skills are even more salient in gifted students, whose higher-order cognitive and emotional needs frequently drive their high performance (Leikin, 2013; Preckel et al., 2006). Therefore, understanding the interplay between mathematical thinking and creativity is critical for optimizing educational practices and fostering cognitive growth across diverse learner groups.

In the modern era, playing digital games is a part of everyday life. Millions of individuals all over the world engage in gameplay. This trend has led to an increased focus on the relationship between digital gaming and higher-order cognitive processes such as mathematical thinking, creativity, and spatial skills in STEM education research (Uttal et al., 2013). The time spent playing digital games, especially strategy or problem-solving genres demonstrates limited benefit for enhancing mathematical thinking and creativity (Qian and Clark, 2016). However, long-term excessive engagement leads to serious problems. The combination of digital games with violent content can negatively impact cognitive performance while diminishing focus and leading to aggressive behavior which then impacts academic results and social adjustment (Ferguson, 2007). Digital games may produce negative effects under conditions of excessive or unguided use that interfere with students' ability to develop complex cognitive functions required for STEM subjects as well as mathematical thinking and creativity. Therefore, this research examined digital game addiction and violent tendencies as mediators between mathematical thinking and creativity specifically for gifted and non-gifted student groups since these cognitive skills are fundamental to STEM fields. Understanding these intricate relationships can serve as a foundation to inform educational practices and intervention strategies within our modern technology-focused society to benefit gifted and non-gifted students.

This study addresses an important gap in the literature by comparatively examining the serial mediating roles of digital game addiction and violent tendencies in the relationship between mathematical thinking and creativity across gifted and non-gifted student groups. While previous studies have primarily focused on isolated cognitive outcomes or general student populations, the present research adopts an integrative framework that simultaneously incorporates cognitive abilities, behavioral risk factors, and digital gaming patterns. Furthermore, by analyzing game genre preferences alongside mediation pathways, the study provides new empirical insights into how different gaming behaviors are associated with distinct cognitive profiles. In doing so, the research offers a more comprehensive and differentiated understanding of how digital gaming contexts interact with higher-order cognitive skills within STEM-related learning environments.

### 1.1 Literature review

#### 1.1.1 Mathematical thinking

Mathematical thinking transforms students into effective problem solvers in the real world by enabling them to acquire solution-focused problem-solving competence, reasoning, and generalization skills through concept learning (Barton, 2011). This critical skill includes abstraction modeling and strategic problem solving that help students construct knowledge, as well as develop reflective thinking and the ability to solve challenging problems (Kooloos et al., 2022). The development of these intellectual skills remains crucial as it enhances students' mental development and their ability to solve problems creatively.

Mathematical thinking comprises various mental processes which consist of problem-solving and reasoning together with communication modeling and representation (Goos and Kaya, 2020). The intricate nature of mathematical thinking demonstrates strong relationships between critical thinking and logical reasoning as well as higher-order thinking, meta-cognition, mathematical modeling, technology integration, and collaborative learning (e.g., Fisher et al., 2018; Lee and Francis, 2018). Students can properly verify assumptions and draw logical conclusions through their development of critical thinking skills (Mulyanto et al., 2018). Through logical thinking, students acquire the capability to build mathematics-related concepts with advanced conceptual understandings (Tajudin and Chinnappan, 2016). The development of analytical reasoning occurs through problem-solving assignments and students achieve higher-order thinking through problem-based learning approaches (Widana et al., 2018). Through the development of meta-cognitive abilities students gain improved methods to control their learning experiences (Suryawan et al., 2023). Mathematical modeling serves as a process to establish links between theoretical mathematical concepts and practical uses in the real world (Haeruman et al., 2024). Education through technology brings new educational instruments that advance mathematics ability and group-learning techniques that let students analyze different perspectives (Andrini, 2023). Multiple evidence indicates that mathematical thinking works through distinct dimensions that require combinations of pedagogical, cognitive, and technical assistance to achieve its highest potential.

The mathematical thinking of gifted and non-gifted students is affected by cognitive skills, educational strategies, and motivational factors (e.g., Divrik, 2023; Efklides, 2018; Trpin, 2024). Gifted students have demonstrated advanced problem-solving skills as well as superior meta-cognitive awareness and creative thinking which surpasses non-gifted student performance (Efklides, 2018). Academic results achieved by gifted students depend as much on their self-efficacy and motivational elements rather than cognitive skills alone. Moreover, several researchers emphasize how supportive educational settings and strategies are needed to increase self-efficacy beliefs in non-gifted students (e.g., Divrik, 2023; Trpin, 2024). Technology also serves as a helpful resource to deliver individualized instruction to gifted students while STEM applications improve their creative and mathematical thinking (Trpin, 2024). Previous studies have indicated that mathematical thinking contributes to problem-solving and creative thinking (e.g., Elgrably and Leikin, 2021; Er et al., 2023; Leikin, 2013; Lithner, 2017). However, the effects of digital game addiction and violent tendencies on this process have not been sufficiently investigated. A closer examination of these factors, differentiating between students with and without giftedness may be useful for comprehending their impact on mathematical thinking and creativity.

#### 1.1.2 The concept of creativity

Creativity is a multidimensional concept that has been approached by different disciplines such as psychology, education, and art. Generally, it is considered the production of new and original ideas, and the capability of assessing and improving constructive ideas (Tubb et al., 2020). According to Pound and Lee (2010), creativity is the generation of ideas plus the analysis and transformation of those ideas, while some processes of creativity do not lead to immediate functional outcomes (Sriraman, 2003). Beyond being a cognitive endeavor, creativity is heavily mediated by social, cultural, and emotional contexts (Kaufman et al., 2010).

Intelligence has usually been correlated with creativity, and long-term research has been carried out in this area. A lower level of intelligence seems necessary for creativity, but this correlation reduces beyond an average level of intelligence (Preckel et al., 2006). That means more environmental factors and individual differences come into play in the actual manifestation of the creativity-intelligence relationship. For example, Fugate et al. (2013) noted that ADHD students generally manifested high levels of creativity in tasks involving original thinking despite their lower working memory. Hence, cognitive diversity plays a role in realizing its potential. Creativity has been fostered in educational settings through meta-cognitive strategies and differentiated teaching methods (Botella et al., 2022); Cetinkaya (2023) further highlights how specially designed educational teaching approaches create essential foundations to develop creativity, especially within gifted student populations.

There are great levels of differences in creativity between gifted and non-gifted (i.e., typically developing) students. Gifted students show consistently higher creative performance than their non-gifted student peers (Wang and Long, 2024). However, aspects such as home environment and emotional intelligence significantly contribute to the development of the creative process as well (Alabbasi, 2024). For instance, the research conducted by Chen and Cheng (2023) showed how emotional intelligence built creative self-efficacy which supported gifted students in their creative work. The research findings also indicated that creativity stemmed from cognitive skills and emotional and environmental stimuli. Although the interplay between mathematical thinking, creativity, and cognitive processes has been studied at great length, the impact digital game addiction and violent tendencies hold over such relations is yet uncharted. Knowing how these factors affect creative thinking processes among gifted and non-gifted students would additionally enrich the information database for the establishment of targeted educational strategies.

#### 1.1.3 Digital game genres

Classifying digital games gives researchers a useful way to understand how children engage with games, as well as how their mental growth, social relationships and emotions develop. Games classifications include content and pedagogical potential. For example, Gee (2003) made a distinction between learning games and entertainment games. The research by Granic et al. (2014) showed entertainment games help improve problem-solving skills. Connolly et al. (2012) proved learning games improve student motivation. Multiplayer games encourage communication and collaboration among children, whereas prosocial games foster positive social behaviors (Lobel et al., 2017). Evaluations of violent games established their ability to boost aggression in children (Gentile et al., 2011). Therefore, game classifications are crucial to analyzing gamers' profiles.

Lowrie and Jorgensen (2011) established a framework featuring six categories that analyzed student preferences for digital games based on corresponding mathematical thinking processes. Players develop their reflexes and motor skills through action games, but adventure games focus on narrative exploration. Through simulation games, students learn basic problem-solving by modeling authentic world scenarios, and they use strategy games to develop strategic thinking skills through logical planning. Role-playing games (RPGs) require players to develop characters as they resolve problems and join the categories with educational and thematic games. Students' cognitive development and mathematical proficiency can be analyzed through the implementation of this analytical framework. The analysis of digital games‘ relationship with mathematical thinking and creativity used the classification framework developed by Lowrie and Jorgensen (2011) as the main theoretical foundation in this research. In addition to mathematical thinking processes, different digital game genres are also closely associated with core components of creativity, such as cognitive flexibility, originality, divergent thinking, and problem restructuring. For example, strategy and role-playing games require players to generate multiple solution pathways, adapt to dynamic problem scenarios, and make innovative decisions under uncertainty, all of which are fundamental characteristics of creative thinking. Similarly, simulation and adventure-based games promote exploratory behavior and imaginative engagement by allowing players to experiment with alternative scenarios and outcomes. Therefore, the framework proposed by Lowrie and Jorgensen (2011) provides not only a cognitive classification grounded in mathematical thinking but also a theoretically relevant structure for examining creativity-related processes within digital gaming contexts.

#### 1.1.4 The mediating role of digital game addiction

It is important to clearly distinguish digital game addiction from game-based learning and recreational digital game use. Digital game addiction is defined as a maladaptive behavioral pattern characterized by impaired control over gaming, increasing priority given to gaming over other life interests and daily activities, and continuation of gaming despite negative academic, social, and psychological consequences (World Health Organization, 2019). In contrast, game-based learning refers to the intentional and pedagogically structured use of digital games to support learning outcomes within controlled educational settings. Therefore, while certain game features may support cognitive engagement under guided and moderate conditions, digital game addiction represents a clinically and behaviorally problematic condition that poses risks to students' cognitive and academic development.

Digital games influence creativity in ways that depend on game genres player characteristics and gameplay (Bulut et al., 2022). The strengthening of problem-solving skills and critical thinking can emerge from digital games while problem-solving and critical thinking form fundamental aspects of creative thinking (Olteteanu and Zunjani, 2020). Minecraft is an example of a simulation and adventure game that promotes creativity since players can create their own worlds and stories (Sotamaa, 2010). The game structure enables players to solve problems creatively since it supports imagination (Zagalo, 2010). Through multiplayer interaction players learn to develop innovative thoughts together while working collaboratively (Takeuchi et al., 2006). Conversely, high-speed game dynamics can degrade attention skills which results in the deterioration of creative thinking processes (Haase and Hanel, 2022). The development of digital game addiction causes decreased creativity through the diminished time individuals spend on additional interests and social and real-world activities (Tarhan and Nurmedov, 2019). Gentile et al. (2011) also established that prolonged gaming leads individuals to become socially isolated while simultaneously lowering their creativity.

Research on digital game addiction's impact on mathematical thinking has shown limited studies while revealing that distractions and focus issues produce adverse effects on mathematical achievement (Benavides-Varela et al., 2020). Conversely, digital games integrate certain features that engage players in mathematical thinking development through problem-solving and critical thinking (Jensen and Skott, 2022). Moreover, digital game-based learning environments provide students the opportunity to experience mathematical concepts while helping them build and construct their understanding of these concepts (Città et al., 2019). However, the risks imposed by digital game addiction necessitate limiting game duration while promoting academic content within digital games. The ambiguous nature of existing research demands extensive additional study which can reveal illuminating insights about digital game addiction alongside its effects on mathematical thinking for developing effective educational strategies.

#### 1.1.5 The mediating role of violent tendencies

The literature provides limited research on mathematical thinking's direct linkage to violent tendencies even though indirect effects and association variables have been documented. Several research findings showed that children who experience bullying tend to achieve unsatisfactory results in mathematics alongside negative school attitudes (e.g., Denson et al., 2011; Vuoksimaa et al., 2021). Moreover, cognitive and emotional traits including low self-control and impulsivity were also linked to violent behaviors (Denson et al., 2011). However, the interplay between these factors and mathematical thinking is not well understood. Creative thinking can serve both constructive and destructive purposes since it exists as a multifaceted connection to violence. Several studies proved that personality characteristics, experiences of social rejection, and negative childhood events significantly shape creative thinking development (e.g., Isacescu et al., 2017; Struk et al., 2017). Creative students became targets of peer bullying according to recent research that showed that poorly performing students sometimes gained social acceptance through bullying behaviors (Faris and Felmlee, 2011). Conversely, Aslan (2023) also indicated how creative thinking skills develop protective factors against violence which subsequently lowers the rate at which gifted students get bullied. Therefore, the mechanism of creativity includes complex functions that show destructive and protective abilities. Research into the effects of mathematics teaching and learning processes on violent tendencies would uncover crucial details regarding their complex relationships.

### 1.2 The current research

The study extends the body of literature on the relationship between mathematical thinking and creativity by comparing this relationship for gifted and non-gifted students. Leikin (2013) states that mathematical problem-solving approaches encourage creative thinking, but there is little research in the literature that specifically relates to this problem and how it differs across different populations of students. As these factors are highly significant in cognitive and emotional development (Gentile et al., 2011), their mediating roles in mathematical thinking and creativity via digital game addiction and violent tendencies necessitate further comprehensive research. Even though academic interest in research on cognitive and creative effects of various digital game genres—such as strategy games, role-playing games, and action games (e.g., Granic et al., 2014; Lowrie and Jorgensen, 2011)—has grown, few if any studies have comprehensively examined the relationship of mathematical thinking and creativity. The examination of gifted students‘ cognitive and creative skills by Preckel et al. (2006) did not generate a sufficient understanding of digital game addiction and violent behavior's influence over these students' development as well as their mathematical thinking and creativity relationship. Therefore, the study investigates how mathematical thinking, directly and indirectly, impacts creativity in gifted and non-gifted students through their digital game addiction behavior and tendency toward violence. The study examines student gaming patterns as well as analyzes how these preferences modify the researched relationships. Consequently, research needs to explore how digital game addiction and violent tendencies function as mediating variables in the relationship between mathematical thinking and creativity because it can allow us to develop specific educational strategies for gifted and non-gifted students.

## 2 Method

The research explored the relationships between mathematical thinking, digital game addiction, violent tendencies, and creativity in gifted and non-gifted students using a multiple mediation model. The research applied a sequential explanatory mixed methods design that emphasized quantitative data analysis yet included qualitative findings for expanded understanding (Creswell, 2013). The qualitative analysis provided more insight and stronger evidence to confirm and elucidate the results obtained from the mediation model in quantitative analysis.

### 2.1 Participants

The research was drawn from two Science and Art Centers for gifted students and six public schools for middle school students (grades 5–8) in a metropolitan area of southern Turkey. The research design used convenience sampling to choose its participant group, which limits generalizability of the research findings. The Ministry of National Education (MoNE) selected the Anatolian Sak Intelligence Scale (ASIS) as a standardized assessment that followed international criteria to identify gifted students between 4 and 12 years old through professional subtests (Sak et al., 2019). Students scoring 130 or above on the ASIS, classified as gifted, receive specialized education at Science and Art Centers in addition to their regular curriculum. Out of 537 questionnaires initially distributed, 29 were excluded due to incomplete responses, resulting in a final sample of 508 students (*M* = 12.54, *SD* = 1.26). This sample comprised 253 gifted students (IQ ≥ 130; 114 girls, and 139 boys; *M* = 12.43 years, *SD* = 1.19; IQ range: 130–157) and 255 non-gifted students (not identified as gifted by MoNE; 134 girls and 121 boys; *M* = 12.67 years, *SD* = 1.23). The total participants consisted of 248 girls (49%) and 260 boys (51%) who were divided into four grade levels comprising 121 (24%) 5^th^ graders and 136 (27%) 6^th^ graders with 132 (26%) 7^th^ graders and 119 (23%) 8^th^ graders. The qualitative stage of study selection involved 34 randomly selected students through cluster sampling (17 gifted and 17 non-gifted) from the quantitative sample pool for interview participation. The gathered interview data enriched the understanding of the study by providing deep insights into its main conclusions.

### 2.2 Measures

#### 2.2.1 Mathematical thinking scale

The research employed the Mathematical Thinking Scale developed by Er et al. (2023) to evaluate participant perception of mathematical thinking skills. This 16-item scale employs a 5-point Likert format (e.g., “Mathematical skills enable me to be more productive in daily life”), with response options ranging from 1 (“Strongly Disagree”) to 5 (“Strongly Agree”). Higher scores indicate stronger mathematical thinking skills, encompassing inductive, deductive, utilitarian, planned, and problem-solving-based thinking based on an individual's views. The scale has demonstrated strong psychometric properties, including excellent internal consistency (α = 0.84), high test-retest reliability (*r* = 0.73), and robust construct validity (χ^2^/*df* = 2.28, CFI = 0.97, IFI = 0.99, RMSEA = 0.06, SRMR = 0.05; Er et al., 2023). In this study, the scale exhibited very good reliability, with a Cronbach's α coefficient of 0.87.

#### 2.2.2 Children's computer game addiction scale

The assessment tool for measuring digital game addiction in students was the Children's Computer Game Addiction Scale which Horzum et al. (2008) developed. Respondents evaluate their gaming behavior by selecting their answers between (1) “Never” and (5) “Always” on a 5-point Likert scale across 21 items on this instrument. Higher points on the scale demonstrate stronger game addiction in children. The scale has obtained psychometric validation with evidence of strong reliability through internal consistency measurements reaching Cronbach's α equal to 0.85. The current investigation yielded high internal consistency from the scale through Cronbach's α equal to 0.86.

#### 2.2.3 Violence tendency scale

Participants' violent tendencies were evaluated using The Violence Tendency Scale developed by Haskan and Yildirim (2012). This 20-item scale is a response on a 3-point Likert-type format (e.g.,“I like to play war-type games on the computer”), with responses ranging from 1 (“Never”) to 3 (“Always”). Their higher scores indicate higher violence dispositions. Haskan and Yildirim (2012) established that the Violence Tendency Scale reached sufficient psychometric qualities because of its strong internal consistency (Cronbach's α = 0.87) and reliable test-retest performance (*r* = 0.83). The current research found high internal consistency reliability for the scale through a Cronbach's α coefficient of 0.88.

#### 2.2.4 Kaufman Domains of Creativity Scale (K-DOCS)

Participants' perceptions of their creativity were assessed by the Kaufman Domains of Creativity Scale (K-DOCS), which was developed by Kaufman (2012). This 42-item scale uses a 5-point Likert format (e.g., “Solving mathematical puzzles”), with responses ranging from 1 (“Much less creative”) to 5 (“Much more creative”). The assessment tool utilizes scale evaluation to measure creative ability perception levels where increased scores point toward stronger creative capabilities. The K-DOCS was adapted for Turkish usage by Sahin (2016) for this research and showed strong scale reliability (Cronbach's α = 0.90) as well as robust construct validity measures (χ^2^*/df* = 1.94, CFI = 0.93, GFI = 0.78, RMSEA = 0.06, SRMR = 0.07; Sahin, 2016). Strong reliability marked the present study's results where Cronbach's α reached 0.89.

#### 2.2.5 The debriefing interview protocol

The research utilized debriefing interview procedures to collect information about students' game-playing behaviors. The debriefing interview technique functions to gather participants‘ consideration regarding particular behaviors and concepts and their attitudes (McAlpine et al., 2002). The researchers directed specific interview questions to students about their digital game activities. The researchers also conducted each interview for about 20 mins to deeply investigate student gaming behaviors.

### 2.3 Procedure and ethics

The quantitative data were collected using the following instruments from the students: Mathematical Thinking Scale, Children's Computer Game Addiction Scale, Violence Tendency Scale, and Kaufman Domains of Creativity Scale (K-DOCS) as well as demographic information (e.g., gender, age, grade, and the weekly digital game-playing time). The data collection took around 40 mins. Then debriefing interviews were conducted with 34 volunteer students to get qualitative data. These students participated in scheduled interview sessions, each lasting around 20 mins, to explore their gaming behavior in depth. According to research ethics, approval was obtained from the university's ethics committee. All students participated voluntarily in the study which was conducted in their classroom at their respective schools.

### 2.4 Data analysis

#### 2.4.1 Quantitative data analysis

A serial mediation analysis was conducted with PROCESS macro for SPSS Model 6 (Hayes, 2017) to evaluate the effect of mathematical thinking on creativity, the relationship between digital game addiction and violent tendencies as well as the effect of violent tendencies on creativity. The research treated digital game addiction and violent tendencies as mediators between variables with gender and age included as covariates. The technique adopted by Van Jaarsveld et al. (2010) enables researchers to understand the singular effect of each mediator in addition to the total sequential effect between both mediators. The mediating variables received assessments through 5,000 bootstrap samples which computed 95% confidence intervals (*CIs*) for establishing indirect effects significance. The result was considered significant when the bootstrap CIs failed to contain zero (Hayes, 2017). Additionally, a 2 × 6 contingency table analysis was performed to evaluate the association between student type (i.e., gifted vs. non-gifted) and preferences for six game genres (Lowrie and Jorgensen, 2011). All data analyses were carried out using IBM SPSS Statistics (version 29.0.1) and JASP (version 0.19.0).

#### 2.4.2 Qualitative data analysis

An interpretative framework was applied to analyze qualitative data, focusing on students‘ digital gaming habits in alignment with the study's objectives (Creswell, 2013). Therefore, the data were collected on students' digital gaming habits. Open and axial coding techniques were employed to analyze the data (Corbin and Strauss, 1990), resulting in the identification and organization of themes and patterns. To ensure credibility and trustworthiness, each researcher independently analyzed the data, focusing on codes, subcategories, categories, themes, and interrelations. Additionally, by applying the credibility and trustworthiness formula proposed by Miles and Huberman (1994), yielding a high credibility coefficient of *p* = 0.94.

## 3 Results

### 3.1 Descriptive statistics and correlations

Table 1 presents the descriptive statistics for the study variables, comparing gifted and non-gifted students. Gifted students demonstrated significantly higher levels of mathematical thinking, creativity, and digital game addiction, whereas non-gifted students exhibited significantly greater violent tendencies. Effect size calculations using Cohen's *d* (Cohen, 1988) indicated that these differences in mathematical thinking, digital game addiction, creativity, and violent tendencies reflected large effect sizes (see Table 1). Moreover, the weekly digital game-playing time of gifted students was 11.47 (± 0.36) hours, while the weekly digital game-playing time of non-gifted students was 9.51 (± 0.48) hours. Subsequent analyses investigated these distinctions, accounting for age and gender as covariates.

**Table 1:** Descriptive statistics for gifted and non-gifted students.

**Construct**	**Gifted students**				**Non-gifted students**				***t-test***	***d***
**Study variables**	***M***	***SD***	***S***.	***K***.	***M***	***SD***	***S***.	***K***.	***t value***	***Cohen's d***
Mathematical thinking	3.95	0.71	−0.67	0.57	3.27	0.61	−0.35	−0.23	9.18^**^	1.02
Digital game addiction	2.95	0.81	0.37	–0.54	2.34	0.70	0.56	0.32	18.77^**^	0.81
Violent tendencies	1.57	0.35	0.57	−0.23	1.92	0.42	1.25	0.60	–7.33^**^	0.90
Creativity	3.37	0.66	0.11	0.46	2.43	0.53	−0.71	−0.25	12.42^**^	1.57

S, skewness; K, kurtosis; ^**^p < 0.001.

Pearson's correlation coefficients were used to determine the relationships between the study variables. Table 2 summarizes these correlations. Gifted students demonstrated a strong and significant positive correlation between mathematical thinking and creativity, whereas non-gifted students exhibited a moderately strong but still significant positive relationship between these two variables. In both groups, mathematical thinking and creativity were negatively correlated with violent tendencies, with the strength of these relationships being moderate to low. For gifted students, digital game addiction showed a moderately positive and significant association with mathematical thinking and creativity. In contrast, non-gifted students showed a weak yet significant negative correlation between digital game addiction and these variables. Additionally, a moderately negative and significant relationship was found between digital game addiction and violent tendencies in gifted students whereas a weak but significant positive correlation was observed in non-gifted students (see Table 2).

**Table 2:** Correlations among the research variables for groups.

**Variables**	**1**	**2**	**3**
1. Mathematical thinking	–		
2. Digital game addiction	*r*(g) = 0.25^*^	–	
	*r*(ng) = −0.18^**^		
3. Violent tendencies	*r*(g) = −0.26^**^	*r*(g) = −0.21^**^	–
	*r*(ng) = −0.13^**^	*r*(ng) = 0.17^**^	
4. Creativity	*r*(g) = 0.62^**^	*r*(g) = 0.27^**^	*r*(g) = −0.27^**^
		*r*(ng) = −0.10^**^	*r*(ng) = −0.16^**^
	*r*(ng) = 0.36^**^		

r(g). Correlation coefficient for gifted student group; r(ng). Correlation coefficient for non-gifted student group; ^**^p < 0.001.

### 3.2 Statistical assumption tests

Before conducting the serial multiple mediation analyses for each group, statistical assumptions were thoroughly evaluated. Univariate normality was assessed by calculating kurtosis and skewness values, which fell within the acceptable range of −1.5 to +1.5, indicating a near-normal distribution for both gifted and non-gifted student groups (George and Mallery, 2003). Reliability coefficients for all variables exceeded the 0.70 threshold recommended by Nunnally and Bernstein (1994), confirming their adequacy. Mahalanobis distance values were below 15 for all cases, suggesting no extreme outliers in either group. Multicollinearity was examined using the variance inflation factor (VIF), tolerance, and Durbin-Watson (DW) statistics. Tolerance values were examined individually for each group, and a holistic analysis across both groups indicated that tolerance values ranged from 0.21 to 0.39, while VIF values varied between 2.17 and 4.63. Furthermore, DW statistics of 1.80 for gifted students and 1.83 for non-gifted students indicated no significant autocorrelation of residuals. These results collectively confirmed the absence of issues related to multicollinearity, linearity, and residual independence, aligning with Field (2016) guidelines for robust statistical analysis in both student groups.

### 3.3 The findings of the serial multiple mediation analyses for each group

**Figure 1:**
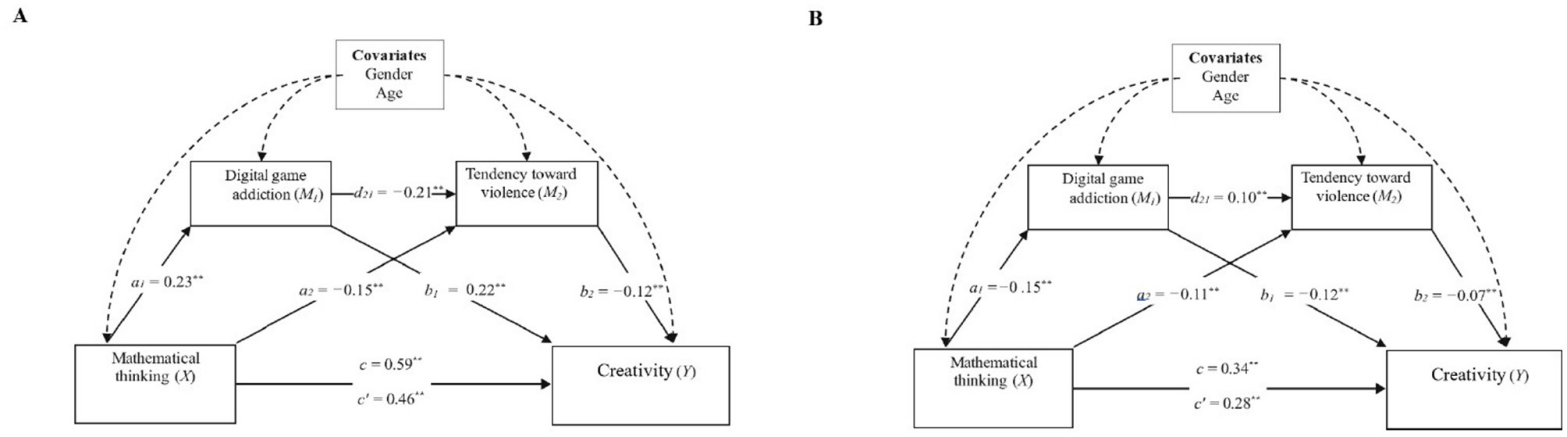
Serial mediation models of the research variables for gifted **(A)** and non-gifted **(B)** groups. Standardized path coefficients are reported. **p < 0.001.

The model was estimated separately for gifted and non-gifted student groups (see Figures 1A, B), and the results confirmed its validity for both groups. The total effect of mathematical thinking on creativity was statistically significant in both models but was notably stronger for gifted students compared to non-gifted students (Gifted: *B* = 0.59, *SE* = 0.17, *t* = 3.55, *p* < 0.001; Non-gifted: *B* = 0.34, *SE* = 0.13, *t* = 2.57, *p* < 0.001). In the gifted group, mathematical thinking had a significantly positive direct effect on digital game addiction (*B* = 0.23, *SE* = 0.06, *t* = 3.88, *p* < 0.001), whereas, in the non-gifted group, this effect was significantly negative (*B* = −0.15, *SE* = 0.05, *t* = –2.88, *p* < 0.001). Similarly, digital game addiction positively influenced creativity in the gifted group (*B* = 0.22, *SE* = 0.08, *t* = 2.86, *p* < 0.001) but had a negative effect in the non-gifted group (*B* = −0.12, *SE* = 0.04, *t* = −2.91, *p* < 0.001).

For both groups, mathematical thinking had a significantly negative direct effect on violent tendencies (Gifted: *B* = −0.15, *SE* = 0.04, *t* = −3.71, *p* < 0.001; Non-gifted: *B* = −0.11, *SE* = 0.04, *t* = −3.01, *p* < 0.001). Additionally, violent tendencies had a significantly negative direct effect on creativity in both groups (Gifted: *B* = −0.12, *SE* = 0.02, *t* = −4.86, *p* < 0.001; Non-gifted: *B* = −0.07, *SE* = 0.03, *t* = −2.74, *p* < 0.001). In the gifted group, digital game addiction was negatively associated with violent tendencies (*B* = −0.21, *SE* = 0.02, *t* = −11.89, *p* < 0.001), whereas in the non-gifted group, this relationship was positive (*B* = 0.10, *SE* = 0.03, *t* = 2.94, *p* < 0.001). When mathematical thinking was modeled alongside the mediating variables (digital game addiction and violent tendencies), its direct effect on creativity diminished in both groups, though it remained statistically significant (Gifted: *B* = 0.46, *SE* = 0.17, *t* = 2.73, *p* < 0.001; Non-gifted: *B* = 0.28, *SE* = 0.12, *t* = 2.28, *p* < 0.001).

According to Table 3, this study revealed that mathematical thinking exerted a significant and stronger indirect effect on creativity in the gifted group compared to the non-gifted group, with digital game addiction serving as a mediating variable [Gifted: *B* = 0.22, *SE* = 0.06, 95% *CI* = (0.10, 0.34); Non-gifted: *B* = 0.14, *SE* = 0.05, 95% *CI* = (0.02, 0.24)]. Violent tendencies slightly but significantly mediated the relationship between mathematical thinking and creativity in both groups [Gifted: *B* = 0.06, *SE* = 0.02, 95% *CI* = (0.01, 0.10); Non-gifted: *B* = 0.04, *SE* = 0.01, 95% *CI* = (0.01, 0.09)]. The serial mediation model, which incorporated both digital game addiction and violent tendencies, also demonstrated a significant indirect effect. For the gifted group, the point estimate was 0.17 (*SE* = 0.06, 95% *CI* = [0.05, 0.29]), while for the non-gifted group, it was 0.10 (*SE* = 0.04, 95% *CI* = [0.02, 0.17]). This model explained approximately 22% of the variance in creativity for the gifted group (*F*_(3, 249)_ = 14.37, *p* < 0.001) and about 10% for the non-gifted group (*F*_(3, 251)_ = 5.78, *p* < 0.001). These findings suggest an indirect relationship between higher mathematical thinking and increased creativity in both groups. In the gifted group, a higher digital game addiction and lower violent tendencies partially mediated this relationship, whereas in the non-gifted group, a lower digital game addiction and lower violent tendencies acted as partial mediators. Moreover, these findings highlighted the nuanced relationships between mathematical thinking, creativity, and the mediating factors in gifted and non-gifted students.

**Table 3:** Results of the separate serial multiple mediation model for each group.

**Path**	**Coefficient**	**SE**	**95% CI**	
			**LL**	**UL**
**Gifted student group**				
M.T. → D.G.A. → C.	0.22	0.06	0.10	0.34
M.T. → T.T.V. → C.	0.06	0.02	0.01	0.10
M.T. → D.G.A. → T.T.V. → C.	0.17	0.06	0.05	0.29
Total effect	0.59	0.17	0.26	0.92
Direct effect	0.46	0.16	0.13	0.79
Total indirect effect	0.43	0.14	0.13	0.72
**Non-gifted student group**				
M.T. → D.G.A. → C.	0.14	0.05	0.02	0.24
M.T. → V.T. → C.	0.04	0.01	0.01	0.09
M.T. → D.G.A. → V.T. → C.	0.10	0.04	0.02	0.17
Total effect	0.34	0.07	0.20	0.47
Direct effect	0.28	0.12	0.14	0.51
Total indirect effect	0.20	0.08	0.05	0.30

C, creativity; D.G.A, digital game addiction; M.T, mathematical thinking; V.T, violent tendencies; SE, standard error; CI, confidence interval; LL, lower limit; UL, upper limit.

### 3.4 Game type preferences for gifted and non-gifted students

A Fisher's Chi-square analysis of the 2 × 6 contingency table examining the relationship between student type (gifted and non-gifted) and game preferences revealed significant results (χ^2^(5, 502) = 106.36, *p* < 0.001). Gifted students showed a strong preference for strategy, adventure, and role-playing games, while non-gifted students favored games in the “other” category including social-casual games (see Table 4). Preferences for action and simulation games were similar across both groups and largely aligned with expectations. These results underscore distinct patterns in game preferences based on student type.

**Table 4:** Observed and expected frequencies of game type preferences for gifted and non-gifted students.

**Game type**	**Observed (gifted)**	**Expected (gifted)**	**Observed (non-gifted)**	**Expected (non-gifted)**	**Key insight**
Action	85	81.18	78	81.82	Similar preferences across groups.
Strategy	37	25.40	14	25.60	Gifted students more prefer strategy games.
Adventure	31	20.92	11	21.08	Higher interest among gifted students.
Simulation	21	20.42	20	20.58	Preferences align with expectations.
Role-playing	68	49.31	31	49.69	Strong preference among gifted students.
Other	11	55.78	101	56.22	Non-gifted students favor this category.

### 3.5 Qualitative results

Student interviews indicated that game type was the primary factor shaping their digital gaming habits, with personal interests and skills playing a significant role in their preferences. Gifted students gravitated toward strategy, adventure, and role-playing games that emphasize problem-solving, strategic thinking, and analytical and creative skills. In contrast, non-gifted students favored social-casual games, such as make-up, dress-up, car-building, and racing games, which prioritize social interaction and psychomotor skills over mathematical reasoning and problem-solving (see Table 5). Gifted students tended to choose digital games purposefully, aligning with their interests to stimulate thinking, reasoning, and creativity, whereas non-gifted students approached gaming as a source of enjoyment, relaxation, and social connection. Furthermore, non-gifted participants showed a stronger preference for social-casual games, where cognitive activities are less prominent. The findings indicated that gifted students engaged with digital games in a deliberate and goal-oriented manner, whereas non-gifted students played more impulsively, prioritizing leisure and social interaction.

**Table 5:** Preference drivers for the selection of digital game type.

**Game type**	**Preference drivers**	**Gifted students**	**Non-gifted students**	**Sample quotes**
Action, Role-playing, Strategy, Adventure, Simulation	Foster creativity, strategic thinking, problem-solving, and analytical skills.	✓	×	“*Minecraft lets me build creatively*.” (GS6), “*Chess challenges my strategy*.” (GS11), “*Mentalup games help me analyze in detail, solve problem and think logically and flexibly*.” (GS14)
Other (social-casual)	Promote social interaction, psychomotor skills, relaxation and enjoyment.	×	✓	“*I made friends on Roblox*.” (NGS9), “*Racing games improve precision and allow me to use the computer mechanically faster*.” (NGS17), “*Dress-up games help me relax and give opportunity me to spare good time*.” (NGS3)

✓. Mostly preferred; × Rarely preferred. GS, Gifted student; NGS, Non-gifted student.

## 4 Discussion

This was a mixed-methods study that provided a detailed exploration of the relationships between mathematical thinking, creativity, digital game addiction, and violent tendencies. Quantitative findings revealed that mathematical thinking was more strongly associated with creativity among gifted students. Notably, digital game addiction was found to function as a significant mediator in the relationship between mathematical thinking and creativity. The relationship between mathematical thinking and creativity was partially mediated by violent tendencies although this effect remained minor in both groups. In the serial mediation model, the indirect association between mathematical thinking and creativity was significant in both groups, but this relationship proved stronger among gifted students when compared to non-gifted students.

The findings from previous studies showed gifted students demonstrated superior performance in problem-solving, abstract reasoning, and creative thinking because of their enhanced cognitive functions and motivational patterns (e.g., Diezmann and Watters, 2001; Ikhwanudin, 2021; Sriraman, 2003). In the context of these studies, gifted students demonstrated better success with challenges at higher difficulty levels and experienced greater achievements through collaborative learning opportunities (Diezmann and Watters, 2001). The stronger link between mathematical thinking and creativity in gifted students might be attributed to the fact that these students exhibited strong mathematical creativity by using metacognitive strategies to generate innovative solutions to complex problems based on previous research. Conversely, Ay and Dogan, 2024 research revealed that non-gifted students who experienced mathematics anxiety failed to solve problems and reduced creative thinking ability resulting in worse academic outcomes. Consequently, this study confirmed previous research results by showing mathematical thinking was positively associated with creativity, and this association appeared stronger among gifted students. (e.g., Diezmann and Watters, 2001; Sriraman, 2003).

Studies have proven digital games as effective educational tools for boosting mathematics outcomes. Voievoda (2024) demonstrated how digital mathematical games can capture current students' attention while playing a central role in mathematical learning success. The competitive structure of these games was associated with higher engagement in creative thinking, which correlated with more dynamic mathematical thinking processes (e.g., Deng et al., 2020; Yeh and Lin, 2018). Moreover, digital mathematics games became essential tools that enable flexible creative problem-solving to develop mathematical competencies (Joung and Byun, 2021). Therefore, digital game addiction was identified as a statistically significant mediator in this relationship. While digital game addiction was a positive and significant mediator in this relationship for the gifted student group, digital game addiction was a negative and significant mediator in this relationship for the non-gifted student group. At this point, the constructive impact of digital games on creative thinking and mathematical skills appeared to differ from the negative associations observed through digital game addiction, which correlated with diminished academic performance and health issues. This situation revealed the necessity of evaluating individual differences.

Importantly, the positive indirect pathway observed for gifted students should not be interpreted as suggesting that digital game addiction itself is educationally beneficial or normatively desirable. Rather, this pattern appears to reflect the interaction between cognitively demanding game characteristics (such as strategy-oriented, problem-solving-based, and complex gameplay mechanics) and the advanced cognitive profiles of gifted learners. Gifted students may be more likely to engage intensively with challenging digital environments that stimulate higher-order thinking processes closely related to mathematical reasoning and creativity. However, digital game addiction remains a maladaptive behavioral condition and must be clearly distinguished from structured educational gaming or moderate recreational use. Therefore, this mediation pathway should be understood as a contextual cognitive interaction rather than as an endorsement of addictive gaming behaviors.

In this context, a portion of the high digital game addiction scores observed among gifted students may be associated not with clinically problematic addictive behaviors, but rather with intensive engagement in cognitively demanding games, sustained task persistence, and high levels of cognitive involvement. This situation may also be related to the limited ability of measurement instruments to clearly distinguish between intensive game engagement and pathological addiction patterns. Therefore, the present findings should be interpreted cautiously, not as normalizing addictive behaviors, but within the framework of the interaction between measurement context and cognitive profiles.

The study found digital game addiction was significantly associated with the relationship between mathematical thinking and creativity, acting as a positive mediator for gifted students. In contrast, the link between mathematical thinking and creativity showed digital game addiction acted as a negative, moderate, and significant mediator for non-gifted students. Results from the study demonstrated opposite digital game addiction influences between student groups. Therefore, digital game use requires limited implementation with proper guidance while considering individual student characteristics and distinct educational groups. Also, the negative mediating effects of digital game addiction in non-gifted students with their detrimental consequences from this finding, while the positive mediating role that digital game addiction played a more beneficial effect in gifted students. In line with this, Haase and Hanel (2022) also revealed that playing digital games initially elicited positive emotions, which also promoted creativity, but excessive gaming had a negative effect, particularly on academic outcomes in mathematics.

The research results showed that both mathematical thinking and creativity were negatively and significantly associated with violent tendencies across gifted and non-gifted student populations, with stronger associations observed in the gifted student group. Despite reaching statistical significance, the mediating role of violent tendencies should be interpreted cautiously, as the small effect sizes indicate limited practical relevance. Therefore, while this variable may function as a statistically detectable pathway, it is not among the primary mechanisms explaining the relationship between the main predictors and outcomes. Cognitive skills demonstrated opposite relationships to violent tendencies according to previous research findings. For example, Windzio (2023) found that mathematically high-achieving students displayed fewer inclinations toward violence. Moreno (2023) showed that creative self-efficacy generated positive social actions against violence and Fahoum et al. (2022) established that creative thinking reduced conflict bias which lowers violence. A study found that experiencing violent media content in the environment led to increased aggressive behavior and restricted creative thinking (Jaruratanasirikul et al., 2009). Muslu et al. (2017) also demonstrated that individuals with low self-esteem exhibited more violent conduct while showing reduced ability to be creative. The current research findings matched those from previous research. A small body of research suggested that highly creative individuals might display violent inclinations when specific circumstances exist. The study of Gino and Ariely (2012) showed that creative minds tend to find rational explanations for unethical conduct resulting in violent outcomes. The research of Lee and Dow (2011) demonstrated that antagonistic personality traits can increase aggressive conduct and stimulate destructive imaginative thinking. The research results from this investigation run counter to these reports.

This study utilized Fisher's Chi-square analysis which indicated significant differences existed between the game preferences of gifted students compared to non-gifted students. The results revealed gifted students played strategy, adventure, and role-playing games whereas non-gifted students played more social-casual games. Both groups exhibited an equal preference for action and simulation games. Digital game addiction showed a negative association with violent tendencies among gifted students whereas it demonstrated a positive relationship with these tendencies in non-gifted students. The qualitative data expanded understanding by disclosing how game genres affect player behaviors. Qualitative findings presented further indicated that game genres influenced digital gaming habits. Gifted students chose digital games that strengthened their creative thinking and problem-solving skills, yet non-gifted students chose games with social interaction and psychomotor-related mechanics. The research confirmed that gifted students show a preference for digital games that offer challenging mental tasks and problem-solving functionalities during gameplay (e.g., Siegle, 2015; Wolfgang and Snyderman, 2021). Li (2024) also discovered that non-gifted students selected games for interaction as well as social rewards because these systems delivered immediate feedback (Alloway et al., 2014). Therefore, the research results matched findings from previous studies. It can be argued that digital game preferences differ between students who vary cognitively and socially based on the research findings. Even more revealing, the findings revealed that digital game addiction has complex links not only to violent tendencies but also to cognitive skills such as mathematical thinking and creativity, comparing these relationships for gifted and non-gifted student groups. Additionally, it can be inferred that digital game preferences exist beyond basic entertainment preferences by revealing deeper cognitive traits and social behavior preferences regarding the findings.

### 4.1 Limitations and further investigations

This research faces two principal constraints because it uses a cross-sectional mixed-method design while studying middle-school and the sample was drawn from a specific geographical and cultural context, which should be explicitly acknowledged as it limits the ability to make causal inferences and the generalizability of the findings to other populations. Future studies need to use longitudinal designs and recruit larger, more diverse samples, as they would help clarify the long-term effects of mathematical thinking on creativity and the mediating roles of digital game addiction and violent tendencies across various ages and demographic groups. Although the findings suggest a positive relationship between mathematical thinking and creativity, the degree to which skills transfer from digital gaming remains debated. The study conducted by Simons et al. (2016) uncovered restricted proof regarding how cognitive abilities developed in digital games can translate into different subject areas which casts doubt on the practical relevance of observed mathematical thinking and creative improvements in this research. This situation highlights a complicated dynamic that existed between digital game addiction and cognitive abilities including mathematical thinking and creativity within gifted and non-gifted student groups. Therefore, a comprehensive analysis regarding changes in both game features and individual cognitive traits is necessary to develop cognitive skills. The full comprehension of digital game addiction mechanisms that involve game types, time spent and addiction severity levels also enables researchers to explain the connection between mathematics thinking and creativity better. In addition, it would be beneficial to find out the impacts of violent tendencies on creativity and under what personality, family, and environmental factors these come into action in digital environments. In particular, such research may lay the groundwork for a new understanding of mathematical thinking and creativity concerning these key features of digital game addiction and violent behavior in terms of developmental pathology. Such research might also inform educational practices that are more responsive to individual differences and that enhance cognitive development in diverse learning environments.

## 5 Conclusion

This study revealed significant distinctions in how mathematical thinking, creativity, digital game addiction, and violent tendencies interacted between gifted and non-gifted students. For gifted students, mathematical thinking was more strongly associated with creativity, with digital game addiction identified as a positive and significant mediator. On the other hand, for non-gifted students, the influence of mathematical thinking on creativity was more moderate, and digital game addiction served as a negative and significant mediator. Violent tendencies were found to have only a minimal effect on these dynamics in both groups. The gaming choices of gifted students included strategy and role-playing games but non-gifted students tended to play social-casual games. These preferences shaped their gaming habits and, in turn, might have influenced their cognitive outcomes. Together, these findings highlighted the intricate relationship between digital gaming and cognitive development, emphasizing the need for educational strategies that account for individual differences.

It is important to emphasize that the positive indirect pathway observed for gifted students should not be interpreted as an endorsement of digital game addiction as an educationally beneficial practice. This finding reflects the interaction between cognitively demanding game characteristics and the advanced cognitive profiles of gifted students rather than the adaptive value of addictive gaming behaviors. Digital game addiction remains a maladaptive behavioral pattern and should be clearly distinguished from structured educational gaming or moderated recreational use. Therefore, the observed mediation effect should be understood as a contextual cognitive interaction rather than a normative educational outcome.

The study advanced theoretical understanding by demonstrating that digital game addiction mediated the relationship between mathematical thinking and creativity in distinct ways for gifted and non-gifted students. Among gifted students, gaming was associated with higher engagement in cognitive and creative processes, aligning with existing theories that emphasize the value of problem-solving and immersive experiences. Excessive digital gaming was associated with hindered cognitive development among non-gifted students, yet again highlighting the unregulated digital engagement risks. These findings highlighted the need for more refined theoretical frameworks that take into account individual cognitive profiles and gaming preferences. Furthermore, the study shed light on the significance of game type in shaping outcomes, suggesting that strategy and role-playing games fostered creativity and analytical thinking, whereas social-causal games lacked the same cognitive benefits.

The research revealed the necessity of establishing educational programs that match individual needs and differences. Strategy and role-playing games were associated with higher levels of creativity and mathematical thinking among gifted students. For non-gifted students, promoting balanced gaming habits and encouraging engagement with games that challenge cognitive abilities could be crucial to minimizing the risks of addiction. Game preferences might also identify students' cognitive strengths which enables educators to design personalized instruction. Collaboration between educators and game designers is critical to developing engaging, educational games that cater to diverse learning needs. Therefore, digital games can operate as effective learning tools when game development targets cognitive development milestones to help all students develop creativity and analytical skills. In conclusion, this study provided critical insights into the relationship between digital gaming and cognitive development, offering a foundation for more effective and inclusive educational practices. By taking individual differences into account, policymakers and educators could take advantage of the potential of digital games to foster creativity and mathematical thinking among varied student populations.

## Data Availability

The raw data supporting the conclusions of this article will be made available by the authors, without undue reservation.
